# Validation of Suitable Reference Genes for RT-qPCR Data in *Achyranthes bidentata* Blume under Different Experimental Conditions

**DOI:** 10.3389/fpls.2017.00776

**Published:** 2017-05-16

**Authors:** Jinting Li, Xueping Han, Can Wang, Wanzhen Qi, Weiyu Zhang, Li Tang, Xiting Zhao

**Affiliations:** ^1^College of Life Sciences, Henan Normal UniversityXinxiang, China; ^2^Engineering Technology Research Center of Nursing and Utilization of Genuine Chinese Crude DrugsXinxiang, China

**Keywords:** *Achyranthes bidentata* Bl., RT-qPCR data normalization, reference genes, selection, gene validation

## Abstract

Real-time quantitative polymerase chain reaction (RT-qPCR) is a sensitive technique for gene expression studies. However, choosing the appropriate reference gene is essential to obtain reliable results for RT-qPCR assays. In the present work, the expression of eight candidate reference genes, *EF1-α* (elongation factor 1-*α*), *GAPDH* (glyceraldehyde 3-phosphate dehydrogenase), *UBC* (ubiquitin-conjugating enzyme), *UBQ* (polyubiquitin), *ACT* (actin), *β-TUB* (*β*-tubulin), *APT1* (adenine phosphoribosyltransferase 1), and *18S rRNA* (18S ribosomal RNA), was evaluated in *Achyranthes bidentata* samples using two algorithms, geNorm and NormFinder. The samples were classified into groups according to developmental stages, various tissues, stresses (cold, heat, drought, NaCl), and hormone treatments (MeJA, IBA, SA). Suitable combination of reference genes for RT-qPCR normalization should be applied according to different experimental conditions. In this study, *EF1-α*, *UBC*, and *ACT* genes were verified as the suitable reference genes across all tested samples. To validate the suitability of the reference genes, we evaluated the relative expression of *CAS*, which is a gene that may be involved in phytosterol synthesis. Our results provide the foundation for gene expression analysis in *A. bidentata* and other species of Amaranthaceae.

## Introduction

Real-time quantitative polymerase chain reaction (RT-qPCR) has become one of the most efficient and powerful techniques to study molecular biology analysis of gene expression and is widely used because of its reproducibility, accuracy, quantity, and sensitivity in gene expression analysis. Although RT-qPCR is a powerful technique, the results of RT-qPCR data analysis are often affected by different variables, such as RNA purity, RNA quantity, DNA contamination, PCR amplification efficiency, and reverse transcription efficiency (Zhu et al., 2013). To control these variables, it is essential to select one or more suitable reference genes as the commonly applied method for normalizing RT-qPCR data (Guénin et al., 2009; Chen et al., 2011; Kundu et al., 2013).

Traditional housekeeping genes that are universally expressed in all cells are often used as reference genes (Xu et al., 2011), such as *GAPDH*, *18S rRNA*, *ACT*, *UBQ*, *EF1-α*, *β-TUB*, and *UBC* (Wang et al., 2014; Xiao et al., 2014). However, these traditional housekeeping genes are not always stably expressed in all species or experiments. Therefore, it is need to select appropriate reference genes that has a consistent level of expression under specific experimental conditions. At present, several studies on the evaluation and validation of reference genes have been carried out for many plant species, such as *Arabidopsis thaliana* (Czechowski et al., 2005; Remans et al., 2008), potato (Nicot et al., 2005), coffee (Barsalobres-Cavallari et al., 2009; Cruz et al., 2009; de Carvalho et al., 2013), rice (Kim et al., 2003; Jain et al., 2006), tomato (Expósito-Rodríguez et al., 2008; Mascia et al., 2010), wheat (Paolacci et al., 2009), pearl millet (Shivhare and Lata, 2016), barley (Burton et al., 2004), *Brassica napus* (Wang et al., 2014), radish (Xu et al., 2012), apple (Perini et al., 2014), rose (Meng et al., 2013), soybean (Jian et al., 2008; Hu et al., 2009), sugarcane (Iskandar et al., 2004), peanut (Jiang et al., 2011), tobacco (Schmidt and Delaney, 2010), banana (Chen et al., 2011), *Buchloe dactyloides* (Li W. et al., 2015), *Salicornia europaea* (Xiao et al., 2014), flixweed (Xu et al., 2016), kiwifruit (Ferradás et al., 2016), pear (Xu et al., 2015), and *Solanum lycopersicum* L. (Fuentes et al., 2016). However, systematic evaluation of the selection of suitable reference genes for RT-qPCR data normalization in *Achyranthes bidentata* under various experiments has not been reported.

*Achyranthes bidentata* Blume (Amaranthaceae) is a perennial herbaceous plant. It grows mainly on the hillsides or roadsides, about 200–1750 m above sea level, and is widely distributed in China, India, Java, and Japan (Yang et al., 2012; Li Y. et al., 2015). *A. bidentata* is one of the most important Chinese traditional medicinal herbs (Liu et al., 2015). It has been frequently used as a diuretic, antipyretic, antirheumatic, and anti-inflammatory drug (Nikolov et al., 1996; Wattel et al., 2004; Li et al., 2005; Jin et al., 2007; Chen et al., 2009; Zhu et al., 2012). In view of its important medical value, the production of *A. bidentata* is gaining more attention. However, the yield of *A. bidentata* is seriously reduced because the growth is influenced by the biotic and abiotic treatment (Li Y. et al., 2015). Some researches on *A. bidentata* are focused at the molecular and biochemical levels. Understanding the expression patterns of some key genes involved in pathway of active components biosynthesis will help understand accumulation and dynamic trends of triterpenoid saponin and ecdysterone in vegetative organs of *A. bidentata*. Furthermore, studies of the molecular events associated with stress responses of *A. bidentata* to multifarious exogenous regulators may also help us understand what causes the loss of *A. bidentata.* At present, RT-qPCR studies in *A. bidentata* are still limited by the use of unsuitable reference genes. Therefore, the selection of the most stable reference genes for *A. bidentata* is essential.

In the present study, we evaluated the stability of eight candidate reference genes (*GAPDH*, *18S rRNA*, *UBQ*, *EF1-α*, *UBC*, *β-TUB*, *APT1*, *ACT*) for normalization across set of biological samples representing *A. bidentata* under different experiments. Two statistical algorithms such as geNorm version 3.5 (Vandesompele et al., 2002) and NormFinder (Andersen et al., 2004) were used to evaluate the most suitable reference genes for a given set of biological samples (Ferradás et al., 2016). To the best of our knowledge, this is the first systematic screening of the appropriate reference gene under a variety of experimental conditions for normalizing gene expression analyses using RT-qPCR in *A. bidentata.* The results will benefit future gene expression analysis in *A. bidentata* and other species of Amaranthaceae.

## Materials and Methods

### Plant Materials and Treatments

This experiment was carried out using the seeds of *A. bidentata*, which were collected from the Wenxian Agricultural Science Institute of Henan Province in China. Seeds were grown in 1 L plastic pots filled with soil (50% nutrient soil, 50% vermiculite sand), and in a greenhouse under growth conditions of 25 ± 2°C, a photoperiod of 14 h, and 20–40% relative humidity. After germination, seedlings were irrigated weekly with Hoagland’s solution.

For samples of six different developmental stages, materials were sampled at the cotyledon, and 1, 2, 3, 4, 5 euphylla stage after germination. The cotyledon, stem, root, and euphylla of seedlings were separated and collected in triplicate, promptly frozen in liquid nitrogen, and finally stored at -80°C.

Material from five separate organs, including cotyledon, euphylla, branch, stem, and root were collected in three replicates, immediately frozen in liquid nitrogen, and stored at -80°C.

Four-week-old seedlings were used to various abiotic and biotic treatments. For cold and heat treatments, plants were grown at temperature of 4 and 42°C for 0, 1, 3, 6, 9, and 12 h, respectively. For drought treatment, plants seedlings were irrigated with 5, 10, and 15% PEG, and leaves were collected at 0, 6, 12, 24, 48 h. For NaCl treatment, seedlings were irrigated with 200 mM NaCl and leaves were collected at 0, 1, 3, 6, 12, 24 h. For hormone treatments, leaves were collected at 0, 1, 2, 3, and 4 days, after seedlings were sprayed with sufficient 1 mg/L MeJA, 1 mg/L IBA, and 3 mg/L SA solutions. The leaves of samples were collected in triplicate, immediately frozen in liquid nitrogen and stored at -80°C until further experiments.

### Total RNA Isolation and cDNA Synthesis

Approximately 100 mg of frozen samples were ground in liquid nitrogen using a pestle and a mortar. Total RNA was extracted from different samples using Trizol reagent (Takara, Japan). A NanoDrop 2000 Spectrophotometer was used to determine the purity and concentration of total RNA. The integrity of the isolated RNA was verified by 1% agarose gel. Only the RNA samples with absorbance ratio at A260/A280 = 1.8∼2.2 and A260/A230 = 2.0 were used for further analysis. For each sample, 1 mg of total RNA was used for first strand cDNA synthesis according to the manufacturer’s instructions (HiScript^®^ 1st Strand cDNA Synthesis Kit, Vazyme, China). The cDNA products diluted with nuclease-free water in the ratio 1:10 were used in RT-qPCR studies.

### Selection of Candidate Genes and Primer Design

The eight common candidate housekeeping genes were selected based on the studies on other species (Czechowski et al., 2005; Jain et al., 2006; Lin and Lai, 2010; Chen et al., 2011; Perini et al., 2014; Wang et al., 2014; Xiao et al., 2014). In this experiment, these genes include: *EF1-α*, *GAPDH*, *UBC*, *UBQ*, *ACT*, *β-TUB*, *APT1*, *18S rRNA*. Sequences of *18S rRNA* was obtained from National Center for Biotechnology Information (NCBI, USA). The sequences of other selected genes were collected from the *A. bidentata* transcriptome database (PRJNA350183), which was obtained by high-throughput Illumina sequencing (Li et al., 2016b).

Specific primers for RT-qPCR analysis were designed using Primer Premier 5.0 software (**Table 1**). A standard curve was repeated in three dependent plates using a 10-fold dilution series ([1/1], [1/10], [1/100], [1/1000], [1/10000]) of the mixed cDNA from all tested samples as the template and it was used to calculate the correlation coefficient (*R*^2^) and gene specific PCR amplification efficiency (*E* = 10^-1/slope^) of each gene (Radonic et al., 2004) (**Table 1**). The specificity of the all primer pairs of candidate reference genes and target gene were verified by agarose gel electrophoresis (3%) and melting curve analysis.

**Table 1:** Candidate *A. Bidentata* reference genes and target genes, primer sequences and amplicon characteristics.

Gene symbol	Gene name	RNA-Seq number	Primer sequence (5′–3′)	Amplicon length (bp)	Amplification efficiency	*R*^2^
*EF1-α*	Elongation factor 1-*α*	UN011764	GAGGCTGCTGAGATGAACAA	166	2.048	0.981
			TGATAAAGTCACGGTGTCCAG			
*β-TUB*	*β*-tubulin	UN053874	GCTTACTTTCTCCGTGTTCC	137	1.944	0.992
			TGTCATAAAGAGCCTCATTGTC			
*ACT*	Actin	UN011760	CCAAGGGCTGTCTTTCCA	80	1.907	0.988
			TAGGCATCCTTCTGTCCCAT			
*18SrRNA*	18S ribosomal RNA		CAGAACATCTAAGGGCATCACA		1.931	0.996
			TAGTTGGTGGAGCGATTTGTCT			
*GAPDH*	Glyceraldehyde 3-phosphate dehydrogenase	UN053070	GGTCACAGGAACCCAGAGG	168	1.807	0.980
			AACGACAAACATTGGAGCATC			
*UBC*	Ubiquitin-conjugating enzyme	UN008343	AGAGGTTGATGCGGGATTTC	101	2.08	0.981
			GATAACGGCATTCCAGAGCA			
*UBQ*	Polyubiquitin	UN005599	CGATTGATAATGTGAAGGCG	167	1.964	0.998
			CTGACCACCACGAAGACGA			
*APT1*	Adenine phosphoribosyltransferase 1	UN001675	GCATGTGGGTGCAGTAGAA	110	2.013	0.995
			GCACTCCTACACGCTCAAG			
*CAS*	Cycloartenol synthase	Contig7815	AGATGTTGAGGGAGAAGGCG	158	1.865	0.993
			GGTCTTCCACCCAACAACAAA			


### Real-Time Quantitative PCR

All reactions were carried out in 96-well optical plates with a LightCycler 96 (Roche, Switzerland) with SYBR^®^Green Master Mix (Vazyme, China). Each reaction mixture contained 0.4 μL of each primer, 10 μL of SYBR^®^Green Master Mix, 2 μL of diluted cDNA (1:10), and 7.2 μL of double distilled water to a final volume of 20 μL. Following cycling conditions were applied: 95°C for 5 min, 40 cycles at 95°C for 10 s and 60°C for 30 s in 96-well optical reaction plates. The melting curve was analyzed at 60–95°C after 40 cycles. All RT-qPCR reactions were carried out in biological triplicates with three technical replicates per experiment, and three no-template controls in every run were included to avoid possible DNA contamination.

### Data Statistics

The expression stability of each reference gene through samples was statistically analyzed using geNorm version 3.5 (Vandesompele et al., 2002) and NormFinder (Andersen et al., 2004), which were used as described in their instructions manual. For these analyses, the mean of the Cq values were changed into relative quantities for genes. The relative quantities of gene were imported into NormFinder and geNorm for further analysis. All other statistical computations were performed in Microsoft Excel 2007.

### Normalization of *CAS*

In order to validate the usefulness of the optimal reference genes in RT-qPCR, the relative expression levels of *A. bidentata* gene, *CAS* is not only a key enzyme gene involved in phytosterol synthesis, but also an important regulatory site for triterpenoid synthesis (Kim et al., 2005; Liang et al., 2009; Zhong et al., 2010). The *CAS* gene was analyzed using the one or two most stable reference genes and the most unstable reference gene. The primer pairs used for the RT-qPCR analysis of *CAS* gene are listed in **Table 1**. The relative expression levels of the *CAS* were assessed according to the 2^-ΔΔCt^ method (Livak and Schmittgen, 2001) under heat treatment. The expression level of pre-treatment samples (controls) was set to a value of 1.

## Results

### Verification of Primer Specificity, PCR Efficiency Analysis

**FIGURE 1:**
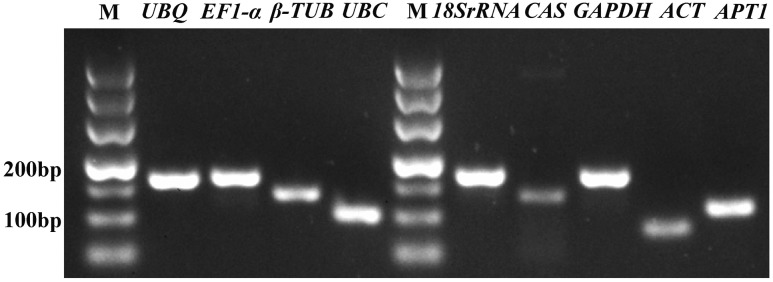
**Specificity of primer pairs for RT-qPCR amplification.** The 3% agarose gel electrophoresis showing a single product of the expected size for each candidate reference gene and the target gene *CAS*; M represents the DNA size marker.

**FIGURE 2:**
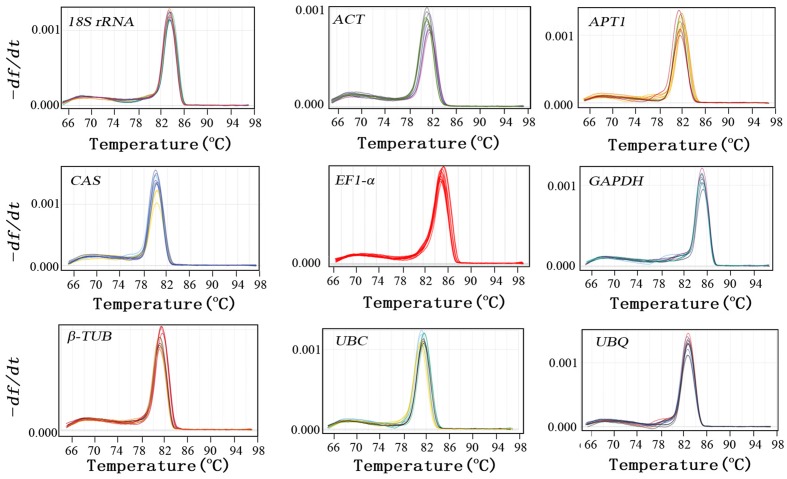
**Specificity of primer pairs for RT-qPCR amplification.** Dissociation curves with single peaks generated for all amplicons.

In order to verify the specificity of the all primer pairs for these candidate reference genes and one target genes, agarose gel electrophoresis (3%) and melting curve analysis were carried out using RT-qPCR. The results of agarose gel electrophoresis (**Figure 1**) showed that all the candidate reference genes were specifically amplified with a very single band of the anticipated fragment size, and primer dimmers or other non-specific amplification products could not be detected on the agarose gel electrophoresis (**Figure 1**). Melting curve analyses of the each internal reference gene after 40 cycles had a single peak (**Figure 2**). These indicated that the eight primer pairs were specific and could be used for the gene expression analysis of RT-qPCR. For each candidate gene, the efficiencies (E) of each primer pair ranged from 1.807 to 2.069 (**Table 1**) and the correlation coefficients (*R*^2^) had a range of 0.971∼0.998 over 10^4^ fold of cDNA dilution (**Table 1**).

### Expression Profile of Reference Genes

**FIGURE 3:**
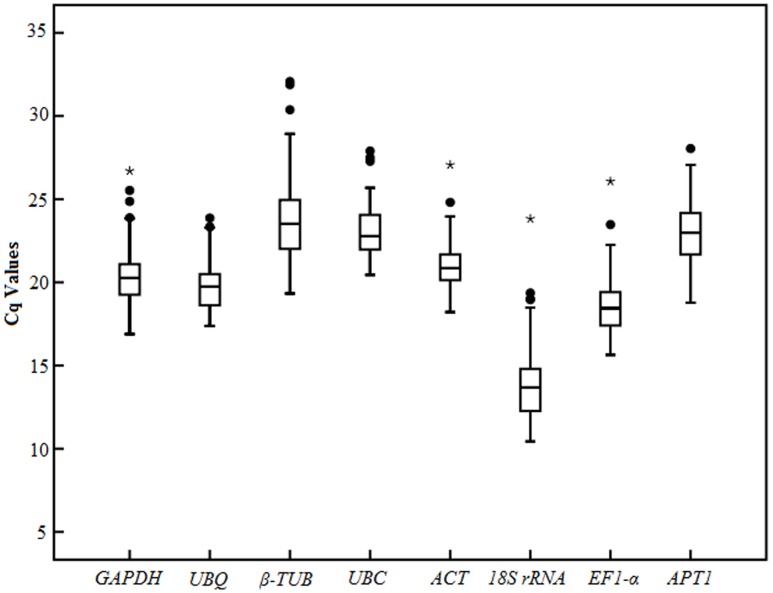
**RT-qPCR raw Cq values for candidate reference genes in different samples.** The box-plot show the mean, maximum, minimum, interquartile range, and outliers.

Analysis of the raw expression levels of each candidate reference gene (*EF1-α*, *GAPDH*, *UBC*, *UBQ*, *ACT*, *APT1*, *18S rRNA*, *β-TUB*) was assessed across all samples (**Figure 3**). In order to obtain reliable results, all RT-qPCR experiments were performed in three technical replicates. The Cq (Bustin et al., 2009) values of the eight reference genes ranged from 10.44 to 28.41 in all tested samples, while the majority of these values were between 18.44 and 23.52 (**Figure 3**). The *UBQ* and *UBC* showed low variability than other candidate reference genes in all the tested samples, with their Cq values ranging from 17.38 to 23.86667 and 20.45 to 27.89667, respectively (**Figure 3**). In addition, *GAPDH*, *APT1*, *β-TUB*, and *EF1-α* showed medium variability, with Cq values of 16.88667–26.7, 18.78–28.045, 19.33–32.075, and 15.64333–26.07, respectively. *β-TUB* and *18S rRNA* indicated high variability, with Cq values of 10.44–23.82 and 19.33-32.075, respectively, while this two genes presented a wider interquartile range than other candidate reference genes in all the tested samples. This indicated that none of the selected genes had a constant level of expression in various tested experiments. Therefore, it is necessary to select suitable reference genes for use in gene expression normalization under different experimental conditions.

### Evaluative of the Expression Stability of Candidate Reference Genes

In order to obtain reliable dataset of the reference genes for 12 groups of samples, two software programs (geNorm and NormFinder) were used to evaluate gene stability. The stability values were determined only in the sample sets of different experimental conditions.

#### GeNorm Analysis

**FIGURE 4:**
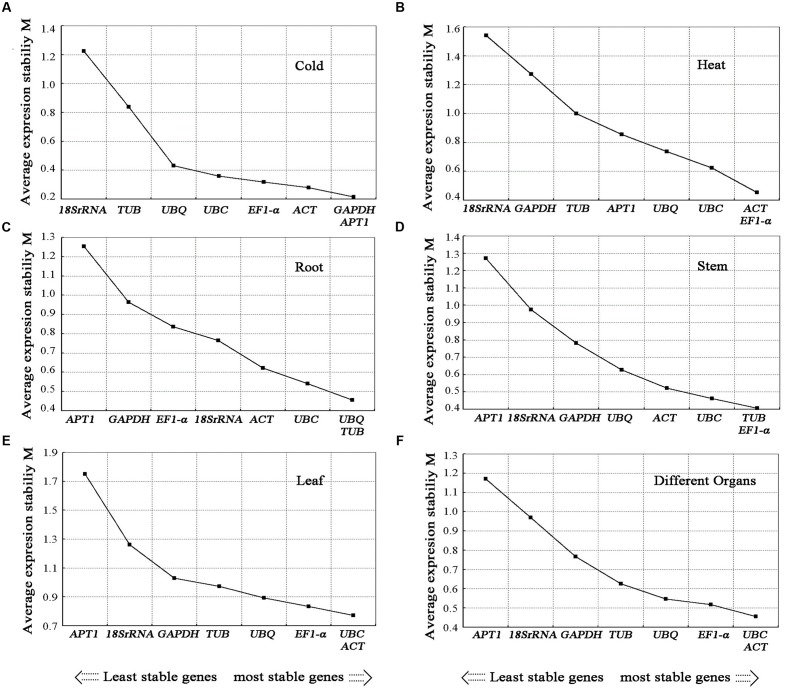
**Average expression stability values (*M*) of candidate reference genes.** Average expression stability values (*M*) of the candidate reference genes were calculated by the geNorm software in *A. bidentata* samples under different experimental conditions, including Cold treatment **(A)**, Heat treatment **(B)**, development stages (Root) **(C)**, development stages (Stem) **(D)**, development stages (Leaf) **(E)**, and different organs **(F)**. The lowest *M*-value indicates the most stable gene and vice versa.

**FIGURE 5:**
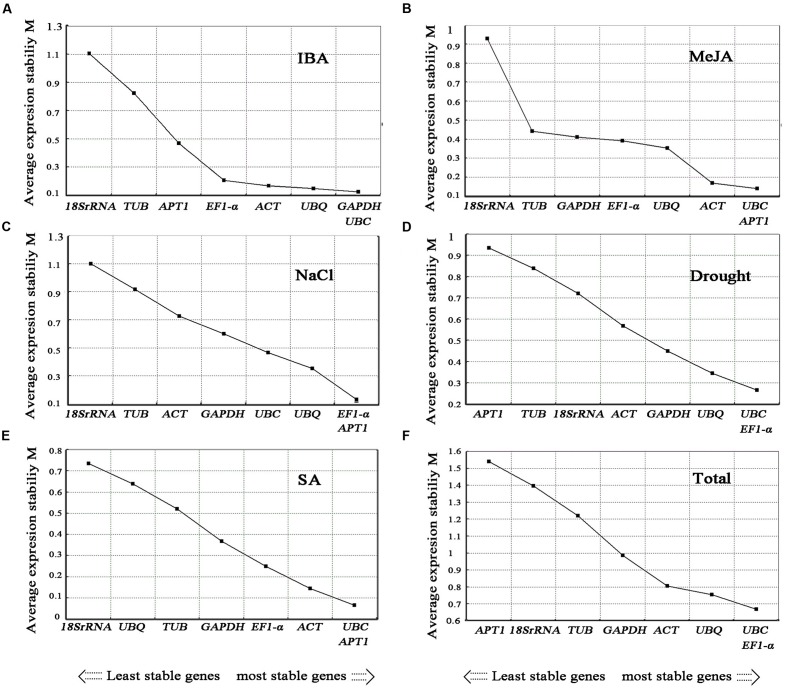
**Average expression stability values (*M*) of the candidate reference genes.** Average expression stability values (*M*) of the candidate reference genes were calculated by the geNorm software in *A. bidentata* samples under different experimental conditions, including IBA treatment **(A)**, MeJA treatment **(B)**, NaCl treatment **(C)**, Drought treatment **(D)**, SA treatment **(E)**, and Total **(F)**. The lowest *M*-value indicates the most stable gene and vice versa.

The gene expression stability measure (M) of the eight genes was the parameter that was assessed using geNorm software (Vandesompele et al., 2002). The Cq values for each cDNA sample was transformed to relative expression levels according to the formula Q = 2^-ΔCt^ (ΔCt = Ct value of each sample – the minimum Ct value), where 2 stand for 100% efficiency (Livak and Schmittgen, 2001; Ramakers et al., 2003) and then evaluated on the basis of the manual. The average expression stability values (M) were calculated at stepwise exclusion of the gene with the least stable reference gene until two best genes were generated. For each group, the gene with the lowest *M*-value was regarded as the most stable expression. Among the eight candidate reference genes used in the analysis, not all the reference genes had constant expression in the different experimental conditions (**Figures 4**, **5**). The *UBC* and *EF1-α* were the two most stable reference genes in all the tested samples. In the different organ sample sets, *UBC* and *ACT* were found to be the most stably expressed. The *UBQ* and *β-TUB*, *β-TUB*, and *EF1-α*, and *UBC* and *ACT* were the best reference genes for normalization in samples at the different developmental stages of roots, stems, and leaves, respectively. For salt treatment, *EF1-α* and *APT1* were evaluated by geNorm as the top two reference genes. In the cold treatment, *GAPDH* and *APT1* were recognized as the most stable genes. The *ACT* and *EF1-α* were suggested as the most stable genes among the eight reference genes in heat treatment. The *UBC* and *EF1-α* genes ranked the highest in the drought treatment. For the MeJA, SA, and IBA hormone treatments, *UBC* and *APT1*, *APT1* and *UBC*, *GAPDH* and *UBC* were chosen as the optimal reference genes, respectively.

**FIGURE 6:**
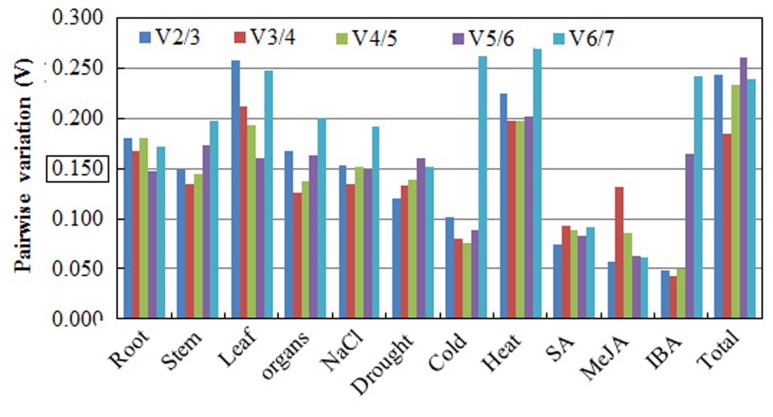
**Pairwise variation (V) analysis of candidate reference genes in the *A. bidentata* sample sets.** The Pairwise variation (V*n*/V*n*+1) between the normalization factors was calculated using the geNorm software program to determine the optimal number of candidate reference genes.

The geNorm software was also used to determine the optimal number of reference genes required for accurate normalization in the different experimental conditions. The Pairwise variation (V*n*/V*n*+1) is an index to determine the minimum number of reference genes required for accurate RT-qPCR normalization in gene expression studies. Vandesompele et al. (2002) recommended using 0.15 as a cutoff value for selecting a suitable number of reference genes, and the additional reference genes below the value of 0.15 are not required for normalization. In our study, the V2/3 values of cold, stem, drought, MeJA, SA, and IBA were less than 0.15, suggesting that the optimal number of reference genes for normalization in these groups was at least two. For different organs and salt stress, the pairwise variation value of V3/4 was 0.126 and 0.134, respectively, suggesting that three reference genes were needed. For the root of different stages, the V5/6 value was 0.147, recommending the selection of five reference genes. However, Pairwise variation analysis (**Figure 6**) indicated that 0.15 is not an absolute cutoff value, but rather an ideal value (Vandesompele et al., 2002; Guénin et al., 2009; Paolacci et al., 2009; Wan et al., 2010). Other studies have shown that Pairwise variation is above 0.15 (V*n*/V*n*+1) for the species under consideration (Silveira et al., 2009). In our study, pairwise variation was higher than 0.15 for all the samples and for different subgroups. Based on these values, we recommended the selection of the top three ranked genes as reference genes for normalization, which is more accurate and reliable than using only one single reference gene.

#### NormFinder Analysis

The NormFinder is an algorithm to select the optimal candidate reference gene. It ranks the full set of candidate genes according to their expression stability in each sample set. NormFinder provides a stability value for each candidate gene, which is a direct measure for evaluating the expression variation when using genes for normalization (Andersen et al., 2004). The lowest expression stability value is the most stable gene.

The results of our analysis using NormFinder program are represented in **Table 2**. Many of the results were consistent with the results of the geNorm analysis. In the samples from different organs and MeJA treatment, *UBQ* and *EF1-α* were the optimal reference gene. Under the cold treatment and developmental stage (leaves), *GAPDH* and *ACT* displayed the best stability value. In the development stages (root and stem), *β-TUB* and *UBC* had the most stable expression. Under NaCl treatment, *EF1-α* and *APT1* showed the common and high stable expression. *EF1-α* and *GAPDH*, *GAPDH* and *UBC*, *ACT* and *APT1* were the two most stable genes under IBA, SA, and heat treatments, respectively. *EF1-α* and *UBC* had the most stable expression in all the tested samples and drought treatment (**Table 2**).

**Table 2:** Expression stability of the candidate reference genes calculated by NormFinder software.

Total		IBA		SA		Cold		Heat		MeJA	
					
Ranking	Stability	Ranking	Stability	Ranking	Stability	Ranking	Stability	Ranking	Stability	Ranking	Stability
*EF 1-a*	0.011	*EF 1-a*	0.004	*GAPDH*	0.010	*GAPDH*	0.008	*ACT*	0.009	*UBQ*	0.004
*UBC*	0.015	*GAPDH*	0.007	*UBC*	0.012	*ACT*	0.008	*APT1*	0.013	*EF 1-a*	0.004
*ACT*	0.031	*UBC*	0.014	*APT1*	0.013	*EF 1-a*	0.010	*EF 1-a*	0.013	*GAPDH*	0.008
*UBQ*	0.035	*ACT*	0.018	*EF 1-a*	0.017	*UBC*	0.012	*UBC*	0.023	*β-TUB*	0.022
*GAPDH*	0.052	*UBQ*	0.019	*ACT*	0.022	*APT1*	0.014	*UBQ*	0.039	*ACT*	0.023
*β-TUB*	0.059	*APT1*	0.038	*UBQ*	0.026	*UBQ*	0.024	*β-TUB*	0.047	*APT1*	0.030
*APT1*	0.078	*β-TUB*	0.062	*β-TUB*	0.029	*β-TUB*	0.069	*GAPDH*	0.104	*UBC*	0.032
*18SrRNA*	0.120	*18S rRNA*	0.118	*18S rRNA*	0.079	*18S rRNA*	0.159	*18S rRNA*	0.132	*18S rRNA*	0.173

**Drought**		**Different organs**		**Root**		**Stem**		**Leaf**		**NaCl**	
					
**Ranking**	**Stability**	**Ranking**	**Stability**	**Ranking**	**Stability**	**Ranking**	**Stability**	**Ranking**	**Stability**	**Ranking**	**Stability**

*EF 1-a*	0.007	*UBQ*	0.015	*UBC*	0.016	*UBC*	0.009	*GAPDH*	0.009	*EF 1-a*	0.007
*UBC*	0.008	*EF 1-a*	0.015	*β-TUB*	0.016	*β-TUB*	0.011	*ACT*	0.011	*APT1*	0.007
*GAPDH*	0.020	*UBC*	0.023	*ACTIN*	0.027	*EF 1-a*	0.011	*UBC*	0.013	*UBC*	0.020
*UBQ*	0.022	*ACTIN*	0.028	*EF 1-a*	0.029	*ACTIN*	0.017	*UBQ*	0.021	*UBQ*	0.025
*ACT*	0.032	*β-TUB*	0.042	*UBQ*	0.044	*UBQ*	0.028	*EF 1-a*	0.025	*GAPDH*	0.039
*β-TUB*	0.046	*GAPDH*	0.046	*GAPDH*	0.055	*GAPDH*	0.060	*β-TUB*	0.039	*β-TUB*	0.056
*APT1*	0.046	*APT1*	0.079	*18s rRNA*	0.060	*APT1*	0.100	*APT1*	0.145	*ACT*	0.058
*18S rRNA*	0.053	*18s rRNA*	0.124	*APT1*	0.093	*18s rRNA*	0.102	*18s rRNA*	0.148	*18s rRNA*	0.093


### Reference Gene Validation

**FIGURE 7:**
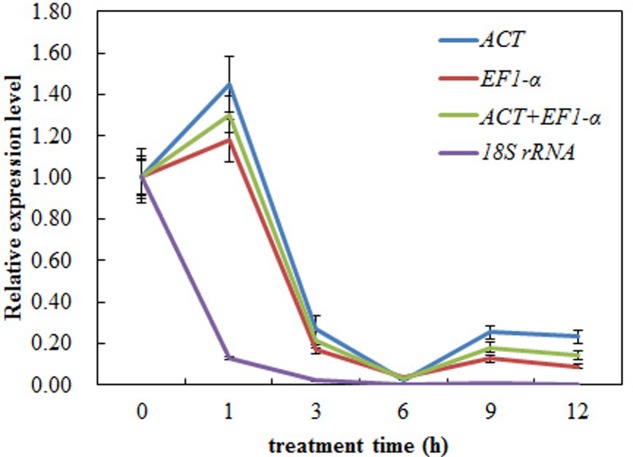
**Relative expression levels of *CAS* using validated reference genes for normalization under the heat stress treatment.** The validated reference gene (s) used as normalization factors were alone (*ACT* and *EF1-α*) or the combination (*ACT* and *EF1-α*) of most stable reference genes, and the most unsuitable one (*18S rRNA*) in heat stress sample set. The expression level of pre-treatment samples (0 h) was set to a value of 1. Each value represents the mean of three replicates, standard error bars are shown.

In order to validate the selected reference gene for normalization, we analyzed the expression of *CAS* using RT-qPCR under the heat treatment. Our results showed that the expression level of *CAS* in leaf decreased obviously during heat treatment and there was only a slight difference between *ACT* and the combination of *ACT*+ *EF1-α* reference gene(s) for normalization (**Figure 7**). However, when *18S rRNA* was used as the most unstable reference gene, the *CAS* gene change patterns were completely changed during normalization under heat treatment (**Figure 7**). These results clearly indicated that the use of improper reference gene could lead to misleading results for the normalization of target gene. Thus, the results of our analysis further emphasized the need of selecting appropriate reference gene stability prior to RT-qPCR study, to avoid low accuracy.

## Discussion

Real-time quantitative PCR as a common technique has been widely used for gene expression analysis in plant species. However, increasing numbers of studies have shown that the selection of unsuitable reference genes could create deviation in results for the expression profile of genes (Gutierrez et al., 2008; Mascia et al., 2010; Wang et al., 2014; Li et al., 2016a; Zhao et al., 2016). Therefore, it is essential to select the appropriate reference gene with the stable expression levels in different experimental conditions in *A. bidentata*.

GeNorm and NormFinder are the two most commonly used software for analyzing the expression stability of candidate reference genes and selecting the most appropriate reference gene sets under certain cases. The two software analysis results are not entirely consistent, probably because of the different statistical algorithms (Hu et al., 2010). Recent studies have shown that analyses in geNorm and NormFinder softwares generate similar results, with only slight difference in ranking orders (Cruz et al., 2009; Lee et al., 2010; Zhao et al., 2016). Our study observed several relevant differences between the two methods as well. However, no matter how the ranking changed, the most unstable gene was the same in both geNorm and NormFinder analyses in all the sample sets, which had been identified based on other species (Artico et al., 2010; Lopez-Pardo et al., 2013; Wang et al., 2014; Li et al., 2016a; You et al., 2016). Therefore, the expression stability value of each gene and the suitable reference genes were obtained by two software packages. Combining the results of the two algorithms together is more reliable when determining the appropriate internal reference genes for normalization.

We evaluated the expression stability of eight candidate reference genes in different organs, during developmental stages and under seven treatments in *A. bidentata*. When all sample groups were evaluated in *A. bidentata*, the three top genes were selected for normalization of gene expression (**Figure 5** and **Table 2**). *EF1-α* was ranked first in both the drought and salt groups. The stability of *EF1-α* in *A. bidentata* was consistent with previous findings in *Solanumly copersicum* L. (Fuentes et al., 2016); however, *EF1-α* is unsuitable for normalization in *Salicornia europaea* under drought stress (Xiao et al., 2014). *UBC* was stable in organ group and different in development stages in root group in *A. bidentata*. Similar stable results have been shown by another group using different tissues in *Salicornia europaea* (Xiao et al., 2014). However, the stability varies by species. For example, *UBC* expressions fluctuated in different organs of bamboo (Fan et al., 2013). *ACT* is satisfactory for RT-qPCR normalization in *A. bidentata. ACT* was highly stable in banana (Chen et al., 2011). However, this gene was not a suitable reference in potato (Nicot et al., 2005). Studies evaluating reference genes in different species have diverse results.

In this study, different sample sets had their own best appropriate internal reference genes (**Figures 4**, **5** and **Table 2**). For instance, *GAPDH* and *ACT* were ranked as the most stable genes in cold treatment, whereas *EF1-α* and *APT1* did better than *GAPDH* and *ACT* under NaCl treatment. For hormone treatments (MeJA and SA), *UBQ* and *EF1-α*, *APT1* and *UBC* were suggested as the most stable reference genes, respectively. For drought treatment in different organs, *EF1-α* and *UBC* were chosen as the most stable reference genes. Our results indicated that specific sets of reference genes are needed in different experimental conditions. Similar results have been obtained in other studies, such as sugarcane (Xue et al., 2014), Jute (Niu et al., 2015), *Cichorium intybus* (Delporte et al., 2015), *Buchloe dactyloides* (Li W. et al., 2015), perennial ryegrass (Lee et al., 2010), and carrot (Campos et al., 2015). It should be pointed out that the most stable internal reference genes were screened out by evaluating eight candidate reference genes that were not frequently used in other plants because there are many reports suggesting that the reference genes are regulated differently in different plants species and they might also show different expression patterns (Maroufi et al., 2010; Chen et al., 2011; Ye et al., 2015; Zhuang et al., 2015). These results highlight the importance of selecting suitable reference gene for each experiment conditions, especially when the samples are very different.

An increasing number of studies have shown that only one reference gene is unsuitable for accurate normalization of RT-qPCR data and the selection of more than one reference genes could get more accurate data in RT-qPCR analysis of plants (Gu et al., 2011). Therefore, the number of reference genes should be considered when a large number of samples need to be analyzed (Lin and Lai, 2010). It is recommended that the number of reference genes to be used, should be determined according to the experimental conditions (Hu et al., 2009). In this work, two or three reference genes were a valid normalization strategy in most experimental cases. For root, five reference genes were needed for normalization. For leaves and heat treatment, we recommend using three reference genes as reference genes for normalization. Our results suggested that selecting a suitable combination of reference genes is necessary according to the different experimental conditions.

To determine the suitability of the reference genes in the present study, we used different reference genes for *CAS* gene normalization. The results showed that the most unstable reference gene for normalization led to significant differences (**Figure 7**). When one gene or the combination genes were used for normalization, the expression pattern of target gene produced slight differences (**Figure 7**). These results suggested that appropriate reference genes are crucial to achieve accurate RT-qPCR results and it was emphasized by the expression analysis of *CAS*. This study provided a foundation for more accurate and widespread use of RT-qPCR in the analysis of gene expression in *A. bidentata.*

## Conclusion

Our results showed that different suitable reference genes for normalization should be selected depending on different experimental conditions. For different sample groups including cold, drought, MeJA, IBA, SA, and the stem of different stages, our study suggested the selection of *GAPDH* and *ACT*, *EF1-α* and *UBC*, *EF1-α* and *UBQ*, *UBC* and *GAPDH*, *APT1* and *UBC*, and *β-TUB* and *UBC*, respectively, for normalizing RT-qPCR data. The *UBC, EF1-α* and *UBQ*, and *EF1-α*, *APT1* and *UBQ* were the three most stable reference genes in the different organs and under NaCl treatment, respectively. For heat treatment, the leaves of different stages and whole samples, *ACT*, *EF1-α* and *UBC* were, respectively, the best reference genes. *ACT*, *EF1-α*, *UBC*, *UBQ*, and *β-TUB* were considered to be the best combination for root of different stages. These results will benefit to the future studies of gene expression in *A. bidentata* using RT-qPCR.

## Author Contributions

JL and XZ designed the project and revised the manuscript. XH, CW, WQ, WZ, and LT were performed the sample collection, analyzed the data and wrote the manuscript. All authors have read and approved the final manuscript.

## Conflict of Interest Statement

The authors declare that the research was conducted in the absence of any commercial or financial relationships that could be construed as a potential conflict of interest.
